# Iron Oxide Nanoradiomaterials: Combining Nanoscale Properties with Radioisotopes for Enhanced Molecular Imaging

**DOI:** 10.1155/2017/1549580

**Published:** 2017-11-21

**Authors:** Juan Pellico, Jordi Llop, Irene Fernández-Barahona, Riju Bhavesh, Jesús Ruiz-Cabello, Fernando Herranz

**Affiliations:** ^1^Centro Nacional de Investigaciones Cardiovasculares Carlos III (CNIC) and Centro de Investigación Biomédica en Red de Enfermedades Respiratorias (CIBERES), 28029 Madrid, Spain; ^2^Radiochemistry and Nuclear Imaging Group, CIC biomaGUNE, Paseo Miramon 182, 20009 Donostia, Spain; ^3^Departamento Química Física II, Facultad de Farmacia, Universidad Complutense de Madrid, 28040 Madrid, Spain; ^4^Centro de Investigación Biomédica en Red de Enfermedades Respiratorias (CIBERES), 28029 Madrid, Spain

## Abstract

The combination of the size-dependent properties of nanomaterials with radioisotopes is emerging as a novel tool for molecular imaging. There are numerous examples already showing how the controlled synthesis of nanoparticles and the incorporation of a radioisotope in the nanostructure offer new features beyond the simple addition of different components. Among the different nanomaterials, iron oxide-based nanoparticles are the most used in imaging because of their versatility. In this review, we will study the different radioisotopes for biomedical imaging, how to incorporate them within the nanoparticles, and what applications they can be used for. Our focus is directed towards what is new in this field, what the nanoparticles can offer to the field of nuclear imaging, and the radioisotopes hybridized with nanomaterials for use in molecular imaging.

## 1. Nanoplatform-Based Molecular Imaging

**Figure 1:**
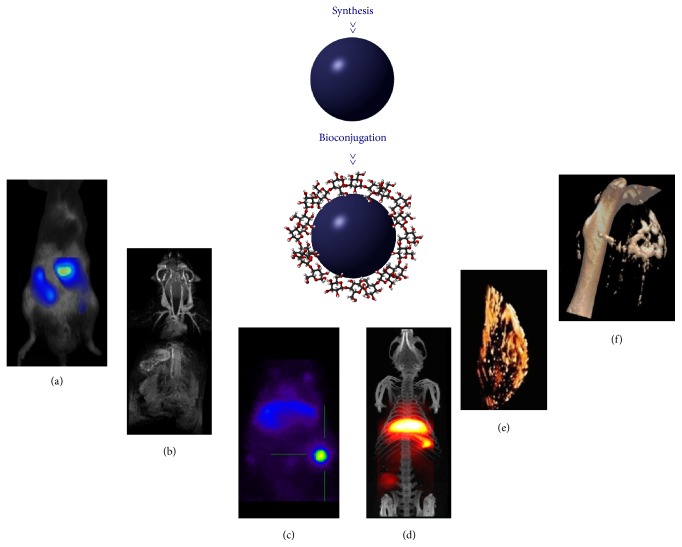
The synthesis and bioconjugation of different nanoparticles enable molecular imaging experiments with different modalities: (a) fluorescence, (b) magnetic resonance imaging, (c) positron emission tomography, (d) magnetic particle imaging/computed tomography, adapted from [18] with permission of the American Chemical Society, (e) photoacoustic imaging, and (f) computed tomography. Panel (d) is adapted from [19], with permission of the Royal Society of Chemistry. Panel (e) is adapted from [20] with permission of Elsevier.

Molecular imaging (MI) is the remote sensing and quantification of the biochemical processes in a living organism at a cellular and molecular level. The interest in the use of nanomaterials (NM) for MI is explained by several factors. Firstly, due to the available variety in composition and size, it is possible to produce probes for every imaging modality (Figure 1). Secondly, the hybrid molecular imaging experiments are much easier to develop due to the intrinsic multifunctional character of most NM. Thirdly, the tailored synthesis of these materials permits fine-tuning the critical parameters like the pharmacokinetics of the probe or the ligand payload. This justifies why most of the NM are used in MI experiments. Examples include, but are not limited to, quantum dots [1, 2], gold nanoparticles [3, 4], upconverting nanophosphors [5, 6], liposomes [7, 8], dendrimers [9, 10], carbon nanotubes [11, 12], silica nanoparticles [13, 14], and perfluorocarbon nanoparticles [15, 16].

The combination of micro/nanoparticles with radioisotopes has long since been used. For example, it is well known that the combination of albumin aggregates with ^99m^Tc for lung perfusion studies [17]. However, the newly developed nanoradiomaterials present a key difference, the precise control over the nanomaterial size (Table 1). The current trend is to combine the size-dependent properties of nanomaterials with the radioisotopes, rather than just using a nanoscaffold.

A particularly important kind of NM in molecular imaging, being the focus here, is iron oxide nanoparticles (IONP). There are many reasons that justify the preferential use of these NM in molecular imaging, like the possibility of getting positive or negative signal in magnetic resonance imaging, tunability of size, the ease of functionalizing their surface, and their biocompatibility or new imaging techniques like magnetic particle imaging [18]. Furthermore, IONP are probably the best example of a new approach in MI, as the combination of nanotechnology and radiochemistry. The combination of the size-dependent properties of nanomaterials and the exquisite sensitivity of nuclear imaging techniques creates a new paradigm in molecular imaging. These new features extend to most of the typical issues when developing tracers for biomedical imaging like the concept of multifunctionality, biodistribution, pharmacokinetics, and the administered dosage.

In this work we will review the synthesis, characterization, and application of iron oxide-based nanoradiomaterials (NRM), focusing on how this is a synergistic approach, beyond the classical attachment of different, independent parts that characterize many multifunctional nanomaterials.

## 2. Iron Oxide Nanoparticles for Molecular Imaging

Iron oxide nanoparticles (IONP) are one of the most used nanomaterials for biomedical applications. They show some remarkable properties explaining their preferential status, such as their size-dependent MRI properties, superparamagnetic behavior, biocompatibility, and chemical stability [24]. They present a magnetic core, typically magnetite (Fe_3_O_4_), maghemite (*γ*-Fe_2_O_3_), or a mixture of both forming a crystalline structure. To avoid aggregation due to surface tension at nanometric scale, magnetic cores are usually accompanied by a coating that reduces surface tension forces, ensuring, hence, the colloidal stability of the sample. The properties of the final formulation depend on the combination of magnetic core and the coating. The selection of the appropriate synthetic method is therefore crucial in obtaining IONP with the desired features [25]. The most commonly used methods are discussed below.

### 2.1. Synthetic Methods

In the last two decades, a variety of synthetic methods have been developed to produce IONP. We can divide the different approaches into two groups: aqueous and nonaqueous methods. Aqueous methods like coprecipitation, hydrothermal synthesis, and sol-gel synthesis produce physiologically stable IONP in a single step. On the other hand, nonaqueous methods produce stable IONP in nonpolar solvents, with better crystallinity and size homogeneity, in comparison with aqueous methods.

#### 2.1.1. Coprecipitation Method

**Scheme 1:**
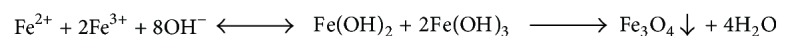
The reaction mechanism of the method developed by Massart.

Coprecipitation is the most used aqueous method to obtain IONP. The first protocol, developed by Massart in 1981, involved the reaction between Fe^2+^/Fe^3+^ salt solutions at basic pH [54]. Under these conditions, with a molar ratio of 1 : 2, ferrous and ferric hydroxides are formed, which finally results in the formation of a Fe_3_O_4_ precipitate (Scheme 1).

Coprecipitation is a straightforward methodology that has been extensively used due to the possibility of producing IONP on a large scale [55]. The main advantage of this method is the production of nanoparticles with colloidal stability in water in a single step. However, the attachment of the surfactant is usually weak, resulting in poor bioconjugation efficiencies.

#### 2.1.2. Thermal Decomposition

Among nonaqueous methods, thermal decomposition of organic precursors is the preferred one. In this method, an organometallic compound together with several surfactants is exposed to high temperatures, leading to decomposition, nucleation, and growth to form the core of the nanoparticle [56, 57]. Organometallic precursors used in this kind of synthesis are Fe(cup)_3_, Fe(CO)_5_, Fe(oleate)_3_, FeO(OH), and Fe(acac)_3_. One of the most successful combinations was proposed by Sun and Zeng in 2002. In this synthesis, Fe(acac)_3_ is heated in diphenyl ether in the presence of oleylamine, oleic acid, and a diol as surfactants [58]. This combination results in highly homogenous and monodisperse, magnetite nanoparticles. The size of the core can be controlled, by adjusting the heating temperature, the reaction time, and/or the concentration of the surfactant, to a range of 3–20 nm. The main advantage of thermal decomposition method is the control over size and shape, due to the fine-tuning of kinetic and thermodynamic parameters. The main drawback of this approach is the hydrophobic character of the nanomaterial that requires a further synthetic step to get water stable nanoparticles. On the other hand, this mandatory second step has also been used for the simultaneous incorporation of hydrophilic character and biological activity with interesting results [59].

#### 2.1.3. Microwave Synthesis

Recently, microwave-driven synthesis has been applied for the development of diverse iron oxide formulations [60–62]. This method offers two key advantages, homogeneous heating and a significantly higher speed of reaction, considerably important for our topic here. In the traditional heat transfer equipment such as heating jackets, oil baths, or sand baths, the sample temperature increases as a consequence of heat exchange through an external source. The process is often slow and the temperature gradients occur within the sample, which implicates local overheating spots. The dielectric heating characteristic of this microwave, on the other hand, prevents temperature gradients and therefore produces highly homogeneous NPs. Furthermore, the use of a microwave oven reduces user dependency while increasing the reproducibility. The usefulness of this approach for biomedical applications has already been proved [60–62].

### 2.2. Iron Oxide for Magnetic Resonance Imaging

**Figure 2:**
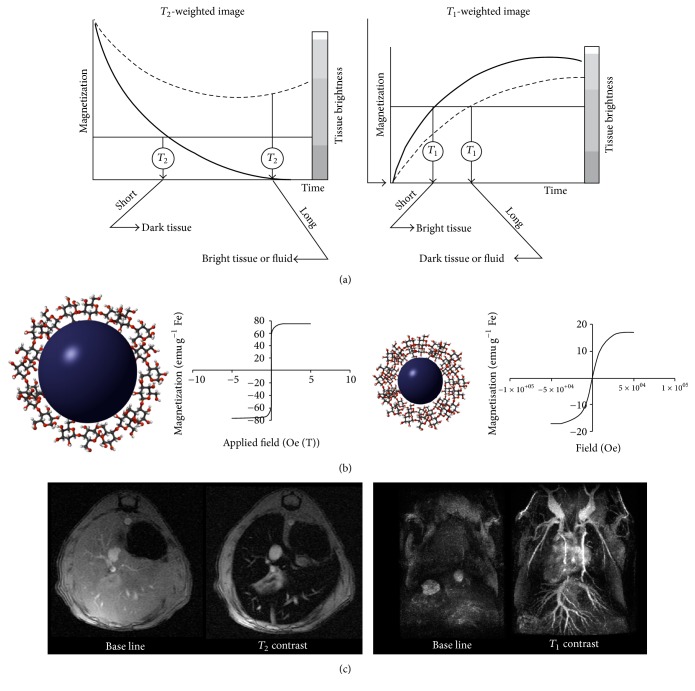
(a) Transversal and longitudinal relaxation times evolution in the presence or absence of contrast agents. (b) Change in the magnetic behavior of iron oxide nanoparticles with the core size, from superparamagnetic (left) to paramagnetic (right). (c)* T*_2_-weighted MRI of the liver using iron oxide nanoparticles with thick organic coating (left),* T*_1_-weighted MR angiography using iron oxide nanoparticles with thin organic coating (right).

Magnetic resonance imaging (MRI) is based on the physical principles of nuclear magnetic resonance (NMR). Briefly, it consists in measuring the evolution of the net magnetic vector generated after placing the sample (the patient) in a large magnetic field. After perturbation of this vector with a radiofrequency pulse, the magnetic vector goes back to the equilibrium state, that is, aligned with the external magnetic field. The recovery of the longitudinal magnetization and the loss of the transversal one are governed by two values,* T*_1_ for the longitudinal and* T*_2_ for the transversal. Contrast agents for MRI reduce both relaxation times; the change of* T*_1_ or* T*_2_ as a function of the concentration of the contrast agent, *r*_1_ or *r*_2_, is what determines the classification of the compound as* T*_1_ or* T*_2_ contrast agent. Large *r*_1_ values brighten the tissue, a “positive” contrast, while very large *r*_2_ values darken the tissue, a “negative” contrast (Figure 2(a)).

IONP are extensively used in the field of biomedicine, as biosensors, in stem cell tracking, magnetic hyperthermia and drug delivery [63–66]. In terms of imaging IONP are well known as contrast agents for magnetic resonance imaging (MRI). IONP are very efficient* T*_2_ contrast agents, due to their superparamagnetic behavior, showing very large *r*_2_ values [67], therefore darkening the tissue surrounding the nanoparticles. However, the diagnosis for many pathologies is complicated by the use of negative contrast agents due to endogenous hypointense signals caused, for instance, by bleeding, metal deposits, or calcifications. In these cases, organs appear completely black, making IONP indistinguishable from endogenous signal. These reasons have, hence, motivated a research interest in finding the methods to obtain IONP for positive contrast agents. We, amongst others, have demonstrated how the use of IONP showing large *r*_1_ values and *r*_2_/*r*_1_ ratios smaller than 3 can be used for T1-MR targeted image [68, 69]. The strategy to develop IONP for* T*_1_ contrast lies in the reduction of the core size down to 2-3 nm. In these conditions, nanoparticles show magnetic behavior much similar to a paramagnetic than to a superparamagnetic compound (Figure 2(b)). Most recently, we have studied the possibility of changing from* T*_2_ to* T*_1_ contrast, by modifying just the thickness of the coating. IONP modified this way show completely different performance* in vivo*, with long circulating times and positive contrast, when the organic coating is thin (about 4 nm), and short circulating times and negative contrast, when the coating layer is thick (about 17 nm, Figure 2(c)), while core size is the same for both (about 4 nm) [70].

### 2.3. Why Still Far from a Clinical Routine Yet?

IONP have long been used in clinic as* T*_2_ contrast agents. Due to the their excretion route, mainly by liver and spleen, these contrast agents are used in the clinic to visualize pathologies in these regions [71–73]. For instance, IONP have been used for diagnosis of hepatocellular carcinoma, colorectal hepatic metastases, pancreatic islet inflammation, and splenic lymphoma [74–77]. However, IONP contrast agents are still far from clinical routine. A variety of iron oxide-based commercial products existed until a few years ago: ferumoxytol (Feraheme®), ferumoxides (Endorem®, Feridex®), ferucarbotran (Resovist®), ferumoxtran-10 (Combidex®, Sinerem®), feruglose (Clariscan®), and ferumoxsil (Lumirem®). The production of some agents such as Sinerem, Feridex, and Clariscan has been discontinued for different reasons, mainly due to regulatory and marketing issues. Currently the only IONP approved for clinical use, Ferumoxytol (Feraheme), was in fact designed as iron supplement for patients with anemia and kidney chronic diseases [78]. After many years of research on the development of IONP, we have a biocompatible, multifunctional nanomaterial with quite a strong signal in* T*_2_-MRI which, however, has not yet found its place in medical imaging within the clinical spectra. Several reasons can be argued to explain this situation, like the hepatic elimination of the particles that produce large accumulations of Fe in the liver, which is a problem especially if repeated doses were necessary [79]. However, in our opinion, the main reason is precisely the claim that its eminent property is the very large negative contrast in MRI. Many times and for most diseases the identification of negative contrast in the image is not straightforward. Anyone who has worked with this kind of material in molecular imaging would agree that being able to differentiate between endogenous hypointense signals and the contrast provided by IONP is nothing but difficult. This difficulty in a clinical environment displaces its usage with Gd-based positive contrast probes, even if this will be at the expense of the inherent toxicity of these compounds. This is one of the most important reasons that explain the interest in combining IONP and radioisotopes. The possibility of combining all the interesting properties of IONP with that of the clear signal from nuclear imaging techniques solves the major part of the problem.

## 3. Radiochemistry for Molecular Imaging

### 3.1. Nuclear Imaging

Nuclear imaging techniques, which comprise positron emission tomography (PET), single photon emission computerized tomography (SPECT), and planar gamma-camera imaging or 2D-scintigraphy, are* in vivo*, ultrasensitive, and minimally invasive imaging modalities which allow the determination of the spatiotemporal distribution of positron- or gamma-emitter labeled tracers (radiotracers) after administration to a living organism. The principle behind PET and SPECT is relatively intuitive: positron emitters undergo spontaneous radioactive decay by emission of a positron, which ultimately interacts with an electron of a surrounding atom in a process called annihilation. This process results in the emission of a pair of gamma rays with energies of 511 keV each and travelling 180° apart. Gamma emitters undergo spontaneous radioactive decay by (*β*^−^, *γ*) emission, through electron capture (EC, *γ*), emission, or isometric transition (IT). When a positron- or gamma-emitter labeled radiotracer is administered to an organism, the high-energy gamma rays escape from the body and are detected by external detectors. All the detected events are finally reconstructed into two-dimensional (2D) or three-dimensional (3D) images, which contain information about the spatiotemporal distribution of the radiotracer within the organism.

Nuclear imaging techniques are extremely sensitive and minimally invasive (the required administration dosage for obtaining an image is limited to a precisely minute quantity of the radiotracer). Hence, repeated studies can be conducted within the same subject over time. The main drawbacks of these techniques are that they require the use of ionizing radiation and the spatial resolution is usually low (in the range of 0.5–1 mm for small animal scanners and a few mm for clinical scanners). Positron emitters typically used in the medical or biomedical fields have shorter half-lives than single photon emitters. Historically, the most commonly used radionuclides have been fluorine-18, carbon-11, nitrogen-13, and oxygen-15, which can be readily produced in small-sized biomedical cyclotrons. Recently, gallium-68 has gained relevance as it can be easily produced in ^68^Ge/^68^Ga generators, which are currently commercially available from different suppliers. However, all these radionuclides have a major limitation: its short half-life, which ranges from 122 s (oxygen-15) to 109.7 min (fluorine-18). Longer-lived positron emitters include zirconium-89 (^89^Zr,* T*_1/2_ = 78.41 h), copper-64 (^64^Cu,* T*_1/2_ = 12.7 h), and iodine-124 (^124^I,* T*_1/2_ = 100.22 h).

### 3.2. Isotopes for SPECT and PET

#### 3.2.1. The Need for Radiolabeling

One of the main drawbacks to consolidating the use of nanomaterials in biomedical applications is the difficulty associated with their tracking, after* in vivo *administration to a living organism. Indeed, the determination of the biodistribution, biological fate, or stability of NPs* in vivo* is extremely challenging. One alternative to gain information regarding the* in vivo* behavior of NPs is to incorporate a radioactive atom into the NP, namely, a positron or gamma emitter. Incorporation of a radionuclide enables the execution of* in vivo* imaging studies using nuclear imaging techniques. The major advantages of radionuclide-based NP tracking are the high sensitivity, the quantitative nature of the detection techniques, and the wide range of radioisotopes available, with different physical properties (see below). However, incorporation of the radioactive atom is usually far from trivial, and the first decision to be made is the selection of the radionuclide. An appropriate selection of the radionuclide requires careful consideration of many different factors, including its physicochemical properties.

As a general rule, the positron or gamma emitters are ideally suited to track NPs* in vivo*, because gamma rays have a high penetration capacity and can easily escape from the body and reach the detectors. However, potential attenuation of the radiation within the organism (which can be significant for low energy gamma emitters and large animal species/humans) may have an effect in the quantification process, and appropriate correction tools need to be considered. Another important factor to consider is the time window in which the NPs should be tracked. If the physical half-life of the radioisotope is too short, the biological process will only be partially investigated. On the other hand, if the physical half-life is too long, high radiation doses might be administered into the organism under investigation and the waste disposal will become more difficult. Finally, the radiochemical integrity of the radiolabeled NP is also paramount. The chemical route to incorporate the radiolabel has to be designed so as to minimize potential loss of the label during subsequent use. Both PET and SPECT rely on the detection of the gamma rays, which are originated in the radioactive atom. If the label (radionuclide) and the NP are not together (due to, e.g., detachment or degradation of the NP), the interpretation of the data will lead to completely wrong conclusions.

With these considerations in mind, the number of positron and gamma emitters that can be principally used for the radiolabeling of NPs is huge. However, in practical terms only a few radionuclides have found real applicability in the field of bionanotechnology. The physical characteristics such as production processes and chemical possibilities for the most relevant positron and gamma emitters in the context of nanomedicine are discussed in the next subsections.

#### 3.2.2. Radiohalogens

Radiohalogens have been used widely for decades due to their well-known chemistry and the wide range of half-lives and emission modes that they offer. Hence, radiohalogens are one of the first options to be considered when approaching the radiolabeling of NPs.

*Fluorine-18*. Fluorine-18 is an accelerator-produced radionuclide, with a decay mode close to 100% positron emission, and it can be generated in two chemical forms (F^−^ and [^18^F]F_2_) by using the ^18^O(p,n)^18^F nuclear reaction. The production of [^18^F]F^−^ is usually achieved by irradiation of ^18^O-enriched water (95–98%) with protons in the energy range of 8–18 MeV, to obtain the radioactive anion as an aqueous solution. The production of [^18^F]F_2_ is based on the so-called “double-shot method” and consists of two irradiation steps. In the first step, pure [^18^O]O_2_ is irradiated with protons to produce ^18^F which remains adsorbed on the walls of the target chamber. The oxygen is then removed by cryogenic retrieval, the target is refilled with a mixture of Ne/F_2_, and a second irradiation is carried out. During the second irradiation, an isotopic exchange reaction between the ^18^F adsorbed on the walls of the target and F_2_ occurs, yielding [^18^F]F_2_.

The main synthetic strategies behind ^18^F-labeling can be crudely divided into two distinct areas. (i) The first area is direct fluorination, where the ^18^F isotope is introduced “directly” into the target molecule of interest in one step via nucleophilic or electrophilic fluorination reactions. Nucleophilic ^18^F^−^ is used to perform aliphatic or aromatic nucleophilic substitution reactions on different leaving groups. This approach is currently used in most of the PET centers worldwide for the daily production of 2-deoxy-2-[^18^F]fluoro-D-glucose ([^18^F]FDG or FDG), a radiofluorinated glucose metabolism marker, which has made an unparalleled contribution in the early diagnosis and evaluation of the response to treatment of a variety of tumors. For electrophilic fluorinations, the most commonly used reagent is [^18^F]F_2_, which can be used directly or converted into less reactive derivatives, such as acetyl hypofluorite (CH_3_COO[^18^F]F), [^18^F]fluoropyridinium [80], and [^18^F]fluoro-N-sulfonamides [81], or other ^18^F-fluorinating N–F reagents such as [^18^F]selectfluor [82]. (ii) The second area is indirect fluorination, which exploits ^18^F-labeled prosthetic groups and is usually applied to radiolabeling of biomolecules which might be unstable under the harsh reaction conditions required for direct fluorination. A large number of ^18^F-labeled prosthetic groups have been developed and applied to ^18^F-fluoroalkylation, ^18^F-fluoroacylation, or ^18^F-fluoroamidation of primary amino groups or thiol residues or used to conduct Huisgen cycloaddition reactions (see [83] for a recent review on ^18^F-fluorination chemistry). Indirect labeling has been applied to the preparation of different ^18^F-labeled NPs [84, 85].

*Radioiodine:*  ^*123*^*I,*  ^*124*^*I,*  ^*125*^*I,  and*  ^*131*^*I*. Iodine-123 is widely used in nuclear imaging; it has a relatively long half-life (13.22 hours) and decays by electron capture to ^123^Te resulting in the emission of gamma rays with a major peak at 159 keV. It is usually produced using the ^124^Te(p,2n)^123^I nuclear reaction using solid, liquid, or gaseous targets, although solid and gas targets are the most commonly used. Radionuclidic impurities can be formed during the production, that is, ^124^I via the (p,n) reaction on ^124^Te or the (p,2n) reaction on ^125^Te [86]. Therefore, the use of ^124^Te with high purity is recommended. When solid targets are used, the irradiated material is elemental tellurium or tellurium oxide [86]. After irradiation, the ^123^I is isolated by distillation and then trapped in a basic solution. Alternatively, the irradiated target can be dissolved in an oxidizing alkaline solution, followed by reduction of the enriched tellurium to the elemental state and iodine to I^−^ with aluminium powder. Precipitated tellurium metal is removed through filtration, and iodide is purified using a cation exchange resin.

Iodine-124 is a positron emitter with a long half-life (4.17 days) and a complex decay scheme, with many high energy *γ*-emissions and high energy positron emission (*E*_*β*max_ = 2.14 MeV, 23% abundance) [87]. It can be produced using different nuclear reactions, with the ^124^Te(p,n)^124^I reaction being currently the most commonly used [86]. For the production, electroplated elemental tellurium or tellurium oxide melted and introduced in the target cavity is used in the solid state. After irradiation, ^124^I is recovered by dry distillation [88] or by dissolution of the irradiated target in an oxidizing alkaline medium followed by reduction of iodine to the I^–^ state by aluminium powder, which can be finally purified by cation exchange chromatography.

Iodine-125 has a half-life of 59.49 days and decays by electron capture to an excited state of ^125^Te, which decays immediately accompanied by emission of gamma rays with a maximum energy of 35.5 keV. Due to the low energy of the emitted gamma rays, attenuation becomes an issue* in vivo* and this radionuclide is mainly used for* in vitro* and ex vivo applications. It is produced in nuclear reactors via the ^124^Xe(n, *γ*)^125m^Xe and ^124^Xe(n, *γ*)^125g^Xe nuclear reactions. ^125m^Xe and ^125g^Xe are unstable and decay to ^125^I with 57 s and 16.9 h half-lives, respectively. After neutron beam, short-lived radionuclides produced during the irradiation are allowed to decay. During this period, the newly created ^125g^Xe becomes ^125^I, which is then collected with aqueous NaOH solution and purified using ion-exchange resins.

Iodine-131 has a half-life of 8.02 days and exhibits 100% decay by electron emission, resulting also in the emission of gamma rays with a maximum of 364.5 keV (81.7%). Due to the simultaneous gamma and *β*^−^ emission, this radionuclide is used in therapeutic applications. It can be produced using two different nuclear reactions, either by neutron irradiation of natural tellurium via the ^130^Te(n, *γ*)^131^Te nuclear reaction (^131^Te decays to ^131^I with a half-life of 25 min) or by irradiation of uranium and chemical recovery from the fission products. Currently, all the above-mentioned radioisotopes of iodine can be obtained from commercial suppliers.

**Figure 3:**
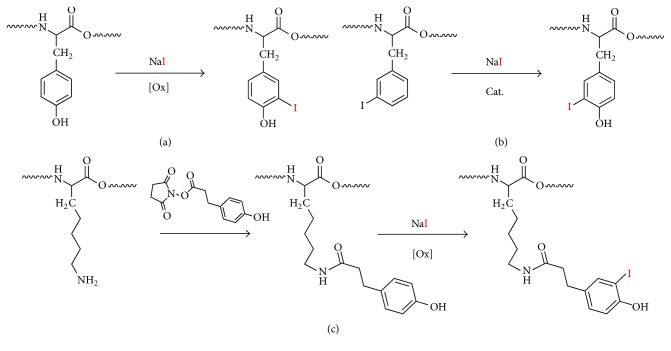
Schematic representation of the main strategies used for the radioiodination: (a) electrophilic substitution, (b) isotopic substitution, and (c) indirect labeling. The red atom represents any radioisotope of iodine.

Radioiodination has been extensively used for decades for the radiolabeling of a wide range of molecular formats, ranging from small molecules to peptides, antibodies, proteins, and nanoparticles. Such labeling can be achieved using mainly three different strategies: (i) In situ oxidation of I^−^ using an oxidizing agent such as* N*-chloro tosylamide or chloramine-T [89], followed by aromatic electrophilic substitution in an activated aromatic ring. This method has been widely used for the radiolabeling of peptides, proteins, and antibodies containing tyrosine residues. As the years pass, more convenient and mild oxidizing agents such as 1,3,4,6-tetrachloro-3*α*,6*α*-diphenyl glycoluril (Iodogen) have been developed [90]. This reagent is insoluble in water and can be deposited on the walls of the reaction vessel, enabling termination of the reaction by removal of the reaction crude. (ii) Indirect methods: They consist of preparing a prelabeled, chemically active group which can be attached to the molecule of interest in a second step. The most widely used conjugation reagent is* N*-succinimidyl 3-(4-hydroxyphenyl) propionate (Bolton–Hunter reagent) [91], which readily forms amides with primary amines. (iii) Isotopic exchange: It is conducted under catalytic conditions. This method requires the presence of a iodine atom in the molecule to be labeled and usually leads to low molar activity values [92]. A summary of the most commonly used strategies is schematized in Figure 3.

Aromatic electrophilic substitution [93] and indirect labeling [47, 94] have been used to date for the preparation of different types of labeled NPs using different radioisotopes of iodine. Additionally, direct adsorption of I^−^ on the surface of certain metallic NPs has also been exploited for imaging purposes [95, 96].

*Radiometals*

**Figure 4:**
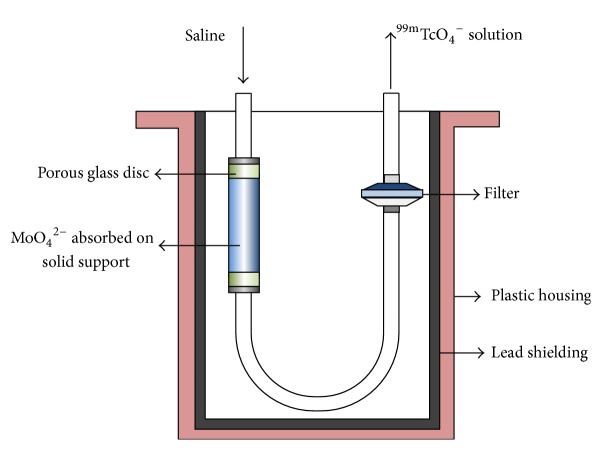
General scheme of a ^99^Mo/^99m^Tc generator.

*Technetium-99m*. Technetium-99m (^99m^Tc) accounts for nearly 80% of nuclear medicine imaging procedures in the clinical field. It has a favourable *γ*-energy (141 keV), a suitable half-life (6.02 h), and well-known coordination chemistry and can easily be obtained as ^99m^TcO_4_^−^ in aqueous buffer from commercially available ^99^Mo/^99m^Tc generators. In such generators, ^99^Mo (*T*_1/2_ = 67 h) is held as ^99^MoO_4_^2−^ on acidic alumina. The ^99^Mo decays forming [^99m^Tc]TcO_4_^−^, which can be eluted on a periodic basis (see Figure 4 for a schematic representation of a standard ^99^Mo/^99m^Tc generator).

The production of the ^99^Mo for the preparation of ^99^Mo/^99 m^Tc generators can be achieved via the ^98^Mo(n, *γ*)^99^Mo nuclear reaction (which leads to low molar activity values) or by irradiation of highly enriched uranium (HEU) with neutrons, which produces the nuclear fission of ^235^U yielding a small fraction of ^99^Mo, the latter being with high molar activity. However, numerous other radionuclides on top of ^99^Mo are produced in this process [97] and the purification is challenging and time consuming [98]. Additionally, a large volume of radioactive waste is generated, and the reactors producing ^99^Mo, having been in operation for a considerable time, need decommissioning or refurbishment [99]. As a consequence, alternative strategies for the direct production of ^99 m^Tc via the ^100^Mo(p,2n)  ^99 m^Tc nuclear reaction have been and are currently being explored [100].

Most of the synthetic methods used in the preparation of ^99m^Tc-labeled radiotracers start from ^99m^TcO_4_^−^, a tetrahedral, d^0^ ion. In aqueous solution, Tc can exist in any oxidation state from VII to I. Ligands that use simple sigma donation from nitrogen, oxygen, and sulphur lead almost exclusively to Tc(V) complexes, which are almost exclusively square pyramidal when the overall charge on the complex is negative or neutral. When the overall charge is positive, the geometry is usually octahedral. Inclusion of mixed ligand systems involving sigma donors and pi acceptors leads to Tc(I), Tc (II), Tc (III), or Tc (IV) complexes. The radiolabeling using ^99m^Tc is based on the formation of complexes, and the synthetic process usually involves TcO_4_^−^, a reducing agent (i.e., Sn^2+^, Fe^2+^, Cu^+^), and a ligand. In the presence of the reducing agent, TcO_4_^−^ is reduced in first instance to a metastable species, which is captured by the ligand. If complexation is weak or slow, TcO_2_ might be formed. In these cases, intermediate complexes with weak ligands can be formed first and the final complex can be achieved by ligand displacement in a second step. In the context of NPs, the most widely used chelator is hydrazinonicotinamide (HYNIC), a complexing agent that acts as a mono- or bidentate ligand, requires coligands such as tricine or N,N′-ethylenediaminediacetic acid (EDDA) to stabilize the radiometal, and has been used to label gold NPs of different nature [101, 102]. Using a completely different approach, superparamagnetic iron oxide nanoparticles (SPIOs) could be labeled using ^99m^Tc-prelabeled bisphosphonates [22].

*Gallium-68 and Gallium-67. *Gallium-68 (*T*_1/2_ = 67.7 min) can be obtained from the parent radionuclide germanium-68 (^68^Ge,* T*_1/2_ = 270.8 days), which decays via electronic capture to ^68^Ga, which subsequently decays to the stable isotope ^68^Zn. Gallium-68 has 89% positron branching and a physical half-life matching the pharmacokinetics of small molecules. This, together with the development of ^68^Ge-^68^Ga generators, which allows daily production of ^68^Ga in ionic form without the need for a cyclotron, has boosted the use of this radionuclide [103]. Before the development of the first ^68^Ge-^68^Ga generator, ^68^Ga was extracted (using liquid-liquid extraction) from irradiated targets in which ^68^Ge was produced via irradiation of natural gallium [104] or gallium/nickel alloys [105]. Due to the unease of this process, the first generators appeared already in the 1960s [106]. In this pioneering work, alumina (Al_2_O_3_) was used to retain ^68^Ge, and ^68^Ga could be eluted from the column using ethylenediaminetetraacetic acid (EDTA) solution. This process has the drawback that the chemical species eluted from the generator is inconvenient to approach subsequent labeling processes; as a consequence, a second generation of “ionic” generators has been developed over the years. Currently, several generators are commercially available, and they differ in the solid support material and the solution used to “milk” the ^68^Ga out of the column. They also show different properties in terms of ^68^Ge breakthrough and presence of metallic impurities. The presence of ^68^Ge and metal ions in the eluted gallium usually requires the implementation of a purification process, which can be achieved by (i) separating the eluate in different fractions and using only those containing the highest concentration of ^68^Ga; (ii) using an anion-exchange resin to selectively trap the anionic chloro complexes of ^68^Ga^3+^ formed under strong acidic conditions (5.5 M HCl) [107], which can be later eluted with small volumes of H_2_O; (iii) selectively retaining ^68^Ga in a cation exchange cartridge by elution using diluted HCl [108]. Metallic impurities can be removed from the cartridge using 80% acetone/0.15 N HCl solution, and elution of the ^68^Ga can be finally achieved by elution with 98% acetone/0.05 N HCl [109].

Gallium-68 has a relatively short half-life that does not match the biological half-life of some macromolecules or NPs. Hence, examples of the use of this radionuclide to label NPs are scarce in the literature [110]. In this context, the use of ^67^Ga might be more convenient. Gallium-67 is a gamma emitter with a long half-life (3.26 d); it decays to stable Zn by electron capture without *β* emission. It can be produced in particle accelerators using different nuclear reactions: ^67^Zn(p,n)^67^Ga, ^68^Zn(p,2n)^67^Ga [111], ^66^Zn(d,n)^67^Ga, and ^67^Zn(d,2n)^67^Ga [112, 113]. Gallium can be finally separated from Zn by ion-exchange chromatography or by liquid extraction [114].

The gallium ion occurring in a solution solely in (III) oxidation state is a hard acid metal and has simple aqueous coordination chemistry. Hence, labeling molecules with ^68^Ga or ^67^Ga is usually performed via chelation. The most widely used chelating agents are 1,4,7,10-tetraazacyclododecane-tetraacetic acid (DOTA), 1,4,7-triazacyclononane-1,4,7-triacetic acid (NOTA), and 1,4,7-triazacyclononane,1-glutaric acid-4,7-acetic acid (NODAGA), all of which are cyclic azacyclo acetic acid compounds. Gallium chemistry is highly influenced by pH change. Labeling at a pH value above 5 is inhibited by formation of stable Ga(OH)_3_, while labeling at very acidic pH values may lead to protonation of the coordinating sites, hence diminishing the coordinative capacity and preventing the formation of the complex. Several examples of radiolabeling of NPs using Gallium-67 and chelators anchored to the NP surface have been described in the literature [115]. Alternative methods based on direct absorption of the Ga^3+^ ion on the surface of metal oxide NPs have also been described (see Section 4.3).

*Copper-64*. Copper-64 has a relatively long half-life (12.7 h) and can undergo electron capture (*ε*, 43.8%), *β*^+^ emission to ^64^Ni (17.8%), and *β*^−^ emission to ^64^Zn (38.4%) [116]. The beta-minus branch along with the emission of Auger electrons makes it an attractive candidate for therapy.

Copper-64 can be effectively produced in nuclear reactors and accelerators. By using reactors, ^64^Cu can be produced via the ^63^Cu(n, *γ*)^64^Cu nuclear reaction by irradiation of stable ^63^Cu (69.1% natural abundance) with thermal neutrons. This leads to low specific activity values. For the production of high specific activity ^64^Cu, fast neutrons can be used, for example, via the ^64^Zn(n,p)^64^Cu nuclear reaction [117]. However, the presence of thermal neutrons, which are always present together with fast neutrons, produces significant quantities of other radionuclides, for example, ^65^Zn (*T*_1/2_ = 243.7 d). Copper-64 can be also produced using cyclotrons via the ^64^Ni(p,n)^64^Cu nuclear reaction. After being first proposed [118], this methodology has been continuously improved. Currently, enriched nickel is first electroplated on a gold disk, which is irradiated with protons. After irradiation, the target material is dissolved in concentrated HCl, and copper and nickel are separated by using an anion-exchange column [119, 120].

Copper-64 exhibits relatively simple chemistry. In solution, it is present in two oxidation states (I and II), and there is a large variety of chelator systems which can form stable complexes with copper [121, 122]. Amongst them, DOTA and NOTA are the most widely used to approach ^64^Cu-radiolabeling. These chelators and others have been used to incorporate ^64^Cu in silica-gold core-shell NPs, [123], quantum dots [124], SWCNTs [125], and monocrystalline IONP [33].

*Zirconium-89*. Zirconium-89 exhibits a decay scheme with 23% positron branching and it has a relatively long half-life (78.4 h). However, the simultaneous emission of a high energy gamma ray with a high branching ratio (99.0%) has negative implications in terms of the effective dose administered into the subject under investigation, limiting its use in certain scenarios. It can be produced by proton irradiation of natural yttrium via the ^89^Y(p,n)^89^Zr nuclear reaction [126], and although radioactive impurities can be formed during irradiation (i.e., ^89m^Zr, ^88^Zr, and ^88^Y), high radionuclidic purity can be achieved using ~15 MeV protons. Separation of ^89^Zr was first achieved by using different solid phase extraction methods [127]. Nowadays, purification is achieved by using an hydroxamate column [128], because, contrary to yttrium, zirconium is able to form complexes with hydroxamates at high acid concentration [129]. With this method, high radionuclidic and radiochemical purities can be achieved with excellent recovery.

In contrast to other radioisotopes previously described, ^89^Zr forms poorly stable complexes with diethylenetriaminepentaacetic acid (DTPA) [130] and ethylenediaminetetraacetic acid (EDTA) [131] and does not fit in chelators such as NOTA or DOTA. In practice, only desferrioxamine (DFO) is currently used in the radiolabeling of biomolecules with ^89^Zr and has been recently applied to the preparation of labeled NPs [132]. However, experimental data suggests that ^89^Zr^4+^ is released from DFO* in vivo*, and hence the development of chelators forming complexes with improved stability is highly desirable. Recently, 3,4,3-(LI-1,2-HOPO) has been suggested as an alternative to DFO with improved chelating properties [133].

## 4. Combined Iron Oxide Nanoparticles and Radioisotopes

### 4.1. What Nanoparticles Change in Nuclear Imaging?

There are several benefits for nuclear imaging, when using nanoparticles, with the most prominent two being pharmacokinetics (PK) and multifunctionality (MF). In the first case, changing the PK of a “traditional” radiotracer is not straightforward, with most of the examples relying on the use of polyethylene glycol chains to, for example, increase the hydrophilicity of the compound (but at the cost of numerous time-consuming steps and reduced radiolabeling yield). The concept of incorporating small molecules in nanocarriers, so that the PK is then that of the nanoparticles, is not new, being one of the key aspects in nanoplatform-based drug delivery. The same idea applies here; upon incorporation of the radioisotope in the NP, the PK is no longer that of the radiotracer, but that of the nanoparticle. Thanks to the nanometric control over the size of the particles and over surface composition the PK can be easily tailored. This allows, for example, obtaining nanoparticles with “à la carte” renal or hepatic excretion. The second aspect, multifunctionality, is an essential aspect of nanoparticles but is seldom found in traditional radiotracers. For example, the synthesis of a nanoradiotracer for PET/MRI/fluorescence and biological activity can be easily done by combining radioisotope core-doped IONP and surface functionalizing with a fluorophore and a peptide [23]. To achieve such complexity with traditional radiotracers would be a tremendous and time-consuming challenge due to the lack of a nanoplatform.

### 4.2. What Radioisotopes Change in Nanoparticles?

Also, here there are several benefits, with sensitivity being the most remarkable one. This applies both to imaging experiments and, as we said before, to the study of nanoparticles' biodistribution. In terms of imaging, the use of nuclear imaging techniques expands, by one or two orders of magnitude, the concentration limit that can be used compared to MRI. Furthermore, when using NP for “negative” contrast in MRI, the identification of the negative signal coming from the nanoparticles is not always straightforward. The incorporation of radioactive signal completely changes this situation. The combined use of PET and MRI helps for much easier localization of the tracer. For the same reasons the study of nanoparticles' biodistribution completely changes upon incorporation of a radioisotope, when it is then possible to quantitatively account for all the injected nanomaterials. These combined advantages explain the large number of already published examples (Table 1). However, a fundamental requisite for this is robust radiolabeling.

### 4.3. Radiolabeling Approaches

There are two important issues to take into account during the development of dual nanoradiotracers based on iron oxide nanoparticles: which radioisotope to take and where to incorporate it within the nanoparticle. The selection of the radioisotope mainly depends on the required half-life time for further imaging experiments and the imaging equipment available. Regarding the localization of the radioisotope, there are two possibilities: either the radioisotope is tagged on the surface of the nanoparticle or it is incorporated directly within the core of the nanoparticle. Irrespectively of the labeling method employed* (vide infra)*, the incorporation of radionuclides into NPs modifies the chemical composition of the NPs. Such modifications can have an effect on the surface of the NPs (e.g., incorporation of a chelate or prelabeled tag, which can ultimately modify the surface properties and zeta potential of the NPs) or the core (when the radionuclide is incorporated by ion/neutron beam or core doping). Thanks to the high sensitivity of nuclear imaging techniques, the mass amount of the radionuclide is usually extremely low (especially if radioisotopes with high specific activity are used). As a consequence, the modifications produced on the NPs are expected to be negligible on a macroscopic scale. In any case, it is convenient to investigate the effect of the labeling process on the physicochemical and biological properties of the labeled particles.

Some of the reported methods for both the approaches, surface labeling and core doping, are listed below.

**Figure 5:**
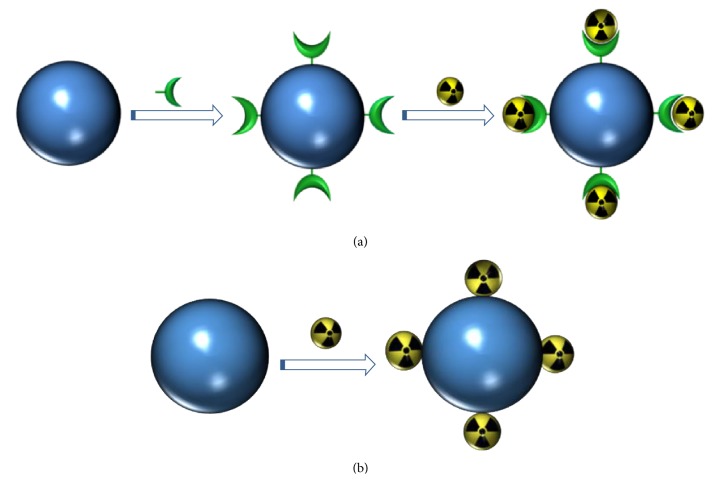
Surface radiolabeling strategies: (a) chelate approach, (b) chelate-free approach.

*(i) Radiolabeling of Nanoparticles on the Surface*. To produce dual iron oxide nanoradiotracers by surface labeling strategies, two approaches can be followed. One of the most used is the chelate approach in which the nanoparticle is functionalized with a ligand able to complex with the radioisotope (Figure 5(a)). A relatively new approach, chelate-free strategy, comprises the addition of the radioisotope to the surface of the nanoparticles without a chelate ligand (Figure 5(b)). In the latter case, the surface must show some affinity towards the radioisotope enabling, hence, the purification and* in vivo* use.

*(i.i) Chelate Approach*. This is a classical strategy where well-known ligands are added to the surface of the IONP to form a complex with the radioisotope.

The development of these probes involves at least three steps: synthesis of IONP, functionalization with the chelate ligand, and finally incorporation of the radioisotope. To select the ligand several features must be considered. First, the ligand should be attached to the surface of the nanoparticles in order to avoid* in vivo* desorption, preferably by covalent bonding. Moreover, the attachment of the ligand should not modify the colloidal properties of the nanoparticles. Finally, the selected ligand must produce a stable coordination complex with the required radioisotope. The stable coordination complex between the radioisotope and the ligand is the key and most problematic point in this method. Once the formulation is intravenously injected, many of the cations present in the bloodstream, like Ca^2+^, Mg^2+^, and Zn^2+^, can trigger a transmetallation reaction, displacing the radioisotope from the coordination complex. If this occurs, the signal recovered in the nuclear imaging equipment comes from the free radioisotope and not from the IONP nanoradiotracer, which may cause a problem in the interpretation of the imaging results.

Among the many ligands studied, a family of cyclic compounds, based on heterocyclic N-dodecane or nonane moieties, has received special attention in the functionalization of the nanoparticles. These ligands, also known as DOTA or NOTA, present tetra or triacetic acids that form a very stable coordination complex with different radioisotopes such as ^64^Cu or ^68^Ga. Macrocyclic ligands are preferred because they present slower dissociation rates than their linear analogous [134]. Examples with this approach have been described for SPECT/MRI or PET/MRI [135, 136]. Concerning SPECT/MRI, ^99m^Tc is the most used radioisotope. Successful radiolabeling is carried out using chelates such as diethylene triamine pentaacetic acid (DTPA) and NOTA. For instance, Madru et al. have describe the synthesis of IONP radiolabeled with ^99m^Tc for the detection of lymph nodes obtaining a 99% of radiolabeling yield [52]. Other SPECT radioisotopes are also used with IONP. Some studies have shown efficient methodologies for the radiolabeling of IONP with longer half-life radioisotopes. Misri et al. produced dual SPECT/MRI probes using DTPA as chelator to incorporate ^111^In-labeled antibody into the IONP [137]. ^125^I has also been used to produce a trimodal SPECT/MRI/Optical imaging probe, based on IONP [138]. Most of examples for PET/MRI probes synthesized by chelator approach use ^64^Cu as positron emitter. DOTA or NOTA is incorporated in the surface of the nanoparticle and then radiolabeled with ^64^Cu. Yang et al. showed a synthesis of IONP radiolabeled with ^64^Cu by coordination with NOTA chelate. They incorporated cRGD into targeted integrins in tumor model and conjugated doxorubicin for drug delivery and treatment of the tumor [35]. Another example uses a DOTA amine derivate chelator to avoid cross-linking side reactions, increasing, hence, the stability of the nanoparticles and radiolabeling yield [139]. Although ^64^Cu is the main radioisotope to produce PET/MRI nanoradiotracers, formulations with ^68^Ga have been already prepared by chelate approach. Kim et al. produced an IONP functionalized with oleanolic acid for tumor targeting and conjugated with NOTA chelate for the radiolabeling with ^68^Ga [39].

Chelate approach is a straightforward synthetic protocol and allows multifunctionalization of the IONP before the radiolabeling, being probably the major advantage of the method. Nevertheless, it is a time-consuming methodology as it requires a multiple-step protocol. This disadvantage has promoted the research on methods to incorporate the radioisotope directly in the surface of the nanoparticle without a chelate ligand.

*(i.ii) Chelate-Free Synthesis*. Chelate-free approach takes advantage of the affinity of some elements towards iron oxide to incorporate the radioisotope directly on the surface of the nanoparticle. It is a relatively new methodology and only a few examples have been reported. For instance, it is know that arsenic presents high affinity towards magnetite [140]. Chen et al. exploited this affinity to incorporate radioactive arsenic into magnetite nanoparticles to produce PET/MRI nanoparticles by chelate-free synthesis [141]. In another example, ^69^Ge is adsorbed on the surface of IONP. This property is frequently used to produce ^68^Ge/^68^Ga generators and it has been used for the radiolabeling of IONP with ^69^Ge on the surface of the nanoparticle [50, 142].

In this approach, the main advantage is that in just one step the radioisotope is incorporated in the nanoparticles. However, there are some inconveniences to be considered. For example, reported examples use radioisotopes that arguably show reduced utility* in vivo,* compared to the radioisotopes employed in other methods. In addition,* in vivo* desorption of the radioisotope from the nanoparticle can occur decreasing signal-to-noise ratio in the imaging experiments and causing toxicity problems in case of arsenic. Recently, Nguyen Pham et al. described the synthesis of chelator-free IONP radiolabeled with PET or SPECT emitters providing available probes for SPECT/PET-MRI [143]. Other examples applied direct radiolabeling of IONP coated with functionalized polyethylene glycol with ^68^Ga resulting in high radiolabeling yields (95%) and good serum stability [144, 145].

**Figure 6:**
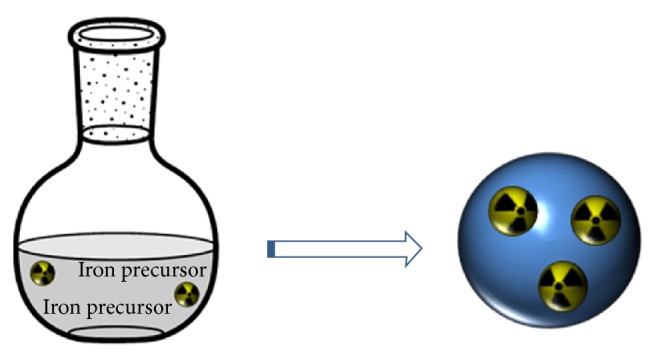
One-step core-doping synthesis of nanoradiomaterials.

*(ii) Radiolabeling of Nanoparticles by Core Doping. *In this method the synthesis of the nanoparticle and the incorporation of the radioisotope are performed simultaneously (Figure 6). Carefully choosing the radioisotopes and synthesis condition permits the incorporation of the isotope within the crystal structure of the iron oxide and not just a simple adsorption or entrapment upon formation of the nanoparticle [23].

Core-doped nanoparticles prevent* in vivo* desorption of the radioisotope from the surface of the nanoparticle and transmetallation reactions, providing excellent radiochemical properties to the nanoradiotracer.

A key point in the core-doping approach is the synthesis technique of the IONP. A fast methodology is required especially if short half-life radioisotope is used for doping. Therefore, reported examples of core-doping approach use microwave-driven synthesis of IONP. As we mentioned before, microwave-driven synthesis allows obtaining IONP in few minutes with highly reproducible results.

Wong et al. reported the first example of core-doped IONP in 2012. They produced IONP coated with dextran and doped with ^64^Cu in 5 minutes using microwave synthesis [146]. They obtained colloidally stable nanoparticles with a modest radiolabeling yield. Most recently, we have described a microwave synthesis of IONP doped with a short half-life isotope, ^68^Ga [23]. We obtained colloidally stable nanoparticles with large radiolabeling yield (~93%) giving an excellent specific activity of 7.6 GBq/mmol Fe, in 15 min total reaction time and complete purification [23]. Nanoparticles, coated with dextran, showed extremely small core size of 2.5 nm and hydrodynamic size around 20 nm. Magnetic characterization revealed* T*_1_ contrast capabilities of the formulation with large *r*_1_ value and modest *r*_2_ value, showcasing this work as the first example of IONP for PET/(*T*_1_) MRI.

*(iii) Radiolabeling of Nanoparticles by Neutron and Ion Irradiation*. Irradiation with accelerated subatomic particles such as neutrons, protons, or deuterons can be used as a general method for the radiolabeling of nanoparticles. This methodology relies on the in situ formation of a radionuclide as a result of a nuclear reaction produced by the interaction of the accelerated particle and one stable isotope present in the NP. This strategy has been used, for example, for the activation of aluminium oxide NPs using proton irradiation [147], the activation of cerium oxide NPs using deuteron irradiation [148], or the activation of gold NPs via the ^197^Au(n, *γ*)^198^Au nuclear reaction [149].

**Figure 7:**
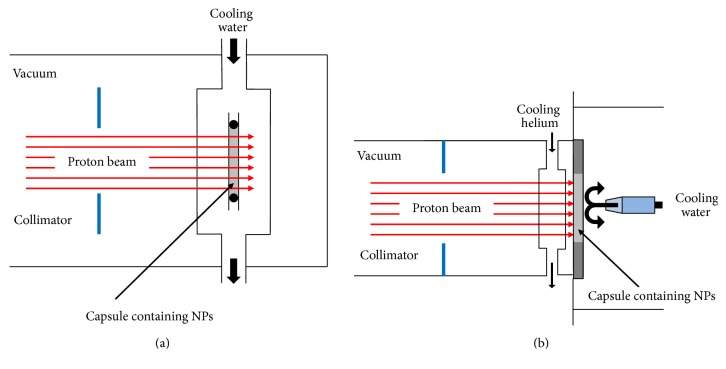
Schematic diagram of the target system used for direct ion-beam activation of NPs using water cooling (a) and water-helium cooling (b). Adapted from [21].

Radiolabeling of NPs by particle irradiation has two main advantages: first, it can be used to activate NPs after their preparation, including industrially produced NPs; second, it can be applied to any NP containing atoms susceptible to nuclear reactions. However, there are two drawbacks that severely limit the applicability of this methodology, requiring careful consideration, and mainly apply to ion irradiation: (i) The activation of one particle is produced by recoil implantation of the radioisotope generated in a different particle. In other words, the radionuclide produced as a consequence of the nuclear reaction travels a few micrometers until its kinetic energy is lost. This means that ion irradiation of NPs can only be carried out in the solid state. When applied to a solution, the radionuclide will have many chances to end up in the solvent. (ii) The nuclear reaction generated by ion irradiation of NPs results in the release of a significant amount of energy, which in turn produces a macroscopic temperature increase limiting the methodology to the activation of NPs that do not contain temperature-sensitive components, for example, organic shells; besides the macroscopic effect, local heating at the nanoscale also requires consideration, because small NPs can be vaporized or promote the formation of aggregates with surrounding particles. Therefore, appropriate cooling during beaming and careful selection of the irradiation conditions are paramount to prevent significant alteration of the physicochemical properties of the NPs. Targets using liquid or gas/liquid cooling have been successfully employed towards the activation of NPs using ion irradiation (Figure 7). The target has to be appropriately designed to ensure effective cooling, especially taking into account the fact that, as mentioned above, NPs in the solid state (powders) need to be irradiated, and the thermal conductivity of powders is usually poor.

In practical terms, the two limitations mentioned above have restricted the application of ion-beam activation of NPs to a few examples in the literature, with all being metal and metal oxide NPs. Due to potential alteration during beam, it is extremely important to investigate potential physicochemical or structural alterations induced on the NPs during beam.

The first example on proton activation of NPs was published by Abbas et al. and described the activation of two types of TiO_2_ which were activated using capsules with different thicknesses, and the effects of the irradiation were evaluated [150]. Local heating on the thick capsules led to phase transition in a fraction of the material. Temperature control of nanoparticulate material by using appropriate target design enabled stable radiolabeling of the NPs without alteration in the state of aggregation [151]. The same strategy was used for the activation of ^18^O-enriched titanium oxide NPs [152]. In this work, the irradiation resulted in the formation of ^18^F via the ^18^O(p,n)^18^F nuclear reaction, although the simultaneous formation of other radioisotopes by activation of titanium, that is, ^48^V, ^47^V, and ^44g^Sc, was observed by high-resolution gamma spectrometry. The radiolabeling of the NPs did not significantly alter the morphological properties of the NPs, as demonstrated by transmission electron microscopy (TEM) and dynamic light scattering (DLS) measurements. Thanks to the presence of ^18^F, short-term* in vivo* biodistribution studies of the metal oxide NPs in rats could be conducted after intravenous and oral administration using PET. The same group also reported the activation of aluminium oxide NPs by proton irradiation via the ^16^O(p, *α*)^13^N nuclear reaction [153]. In this work, different-sized NPs were introduced in an aluminium capsule and irradiated with cyclotron-accelerated protons at 5 *μ*A, to produce ^13^N-labeled NPs. Despite the short half-life of ^13^N (9.97 min), the activation was sufficient to determine the biodistribution pattern after intravenous injection in rodents up to 1 hour. The authors could establish a relationship between the nominal size of the NPs and the organs where accumulation of the NPs took place.

Direct activation of carbon-based NPs by proton irradiation can also be achieved, although relatively high energies (>29 MeV, which cannot be achieved by most of the biomedical cyclotrons) are required. Activation occurs via the (p,3p3n) reaction leading to the creation of ^7^Be. By using protons of energy above 35 MeV, it has been shown that specific activities of some hundreds of kBq/mg can be obtained in carbon black and carbon nanotubes in a reasonable irradiation time [154]. However, the effects of the irradiation on the physicochemical properties of the irradiated material were not investigated.

Proton irradiation can also be employed to the activation of iron oxide NPs, because the most abundant stable isotope of iron (^56^Fe, natural abundance of 91.75%) can undergo the ^56^Fe(p,n)^56^Co nuclear reaction in the energy range 5–30 MeV [155], with ^56^Co being a positron emitter with a half-life of 77.2 days. This nuclear reaction has been employed for the activation of Fe_3_O_4_ (magnetite) NPs using accelerated protons with energy in the range 12–14 MeV [156]. Although other nuclear reactions may occur during proton irradiation, that is, ^57^Fe(p,2n)^56^Co and ^58^Fe(p,3n)^56^Co, their contribution to the overall formation of ^56^Co can be neglected at the above-mentioned proton energies due to the low reaction cross sections and the low natural abundances of ^57^Fe and ^58^Fe (2.2% and 0.28%, resp.). X-ray diffraction (XRD), dynamic light scattering (DLS), and zeta potential measurements before and after activation demonstrated that no changes took place in the crystalline structure, and no alterations were observed in terms of size and zeta potential.

If the material of the NPs cannot be activated by proton irradiation, other accelerated ions might be used, that is, deuterons. Deuteron irradiation was used to create ^141^Ce-labeled NPs via the (d,p) reaction from ^140^Ce [148]. Low beam currents (2 *μ*A) were used to prevent thermal damage of the NPs. DLS and zeta potential measurements confirmed negligible structural and morphological changes in the NPs due to the irradiation procedure.

As mentioned above, irradiation with accelerated ions can pose severe damage to the irradiated particles. As an alternative, neutron irradiation can be used. Neutron activation is mainly performed by exposing the NPs to the intense neutron flux of a nuclear research reactor, resulting generally in (n,*γ*) or (n,p) nuclear reactions, although the latter presents lower cross section values (and hence is less efficient). The yield for neutron capture by (n,*γ*) reactions depends on the neutron energy; in general, the reaction cross section rises as the neutron energy is reduced, following a good approximation of *E*_*n*_^−1/2^ up to energies of about 10^3^ keV, but the cross section value at a given energy can vary several orders of magnitude from one atomic species to another. In practical terms, only a few atoms can undergo neutron activation, including among others ^151^Eu, ^165^Ho, ^187^Re, and ^197^Au, which yield ^152^Eu, ^166^Ho, ^188^Re, and ^198^Au, respectively. Other atoms like Fe can be also activated, but cross section values are much lower. In contrast to ion irradiation, in neutron activation the neutron capture occurs preferentially at low neutron energies, and it can generally be assumed that the activated nucleus remains very close to its original position. As a consequence, irradiation of NPs in solution is feasible with little risk for the radionuclide to end up in the solvent [21], and hence cooling of the irradiated material is more effective than in ion activation, in which powders are usually irradiated.

**Figure 8:**
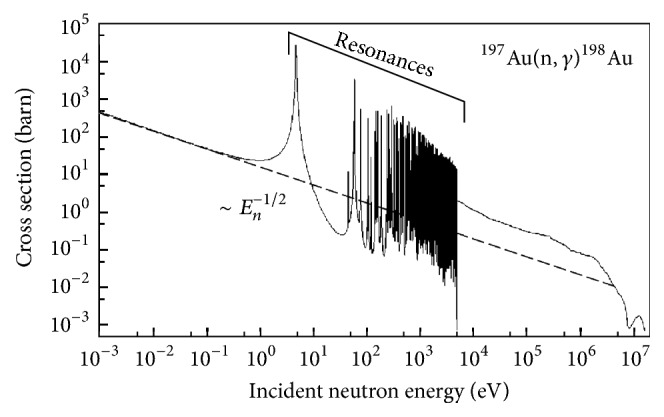
Excitation function for the neutron capture reaction ^197^Au(n,*γ*) ^198^Au. The oscillations of the reaction cross section in the energy range between 5 and 5000 eV are referred to as resonances (data from JEFF 3.1.1 (OECD-NEA 2009)).

One of the main limitations of neutron activation is the need to conduct activation in a nuclear research reactor. To mitigate this drawback, alternative methods have been developed, with one of them being the use of accelerators taking advantage of the adiabatic resonance crossing (ARC). This method relies on gradually slowing down fast neutrons emitted from ion-induced nuclear reactions with high neutron yield. If the neutron moderator is properly designed, the energy of the neutrons can be tuned to match the energy range in which target atoms exhibit resonances in their neutron capture (resonances are energy ranges in which the probability for the neutron capture to occur is very high; see Figure 8 for gold). In this way, the small neutron flux (compared to that obtained in a nuclear reactor) can be compensated by making more efficient use of the neutron-energy regime. Experimental setups have been tested at different locations including CERN [157], Louvain-la-Neuve [158], and JRC Ispra [159] with promising results.

There are a few examples in the literature describing neutron activation of NPs. The preparation of ^166^Ho-labeled holmium acetylacetonate NPs could be achieved by neutron activation using neutron flux of 5 × 10^12^ cm^−2^ s^−1^ during 60 minutes. In this case, specific activities of 12 MBq/mg could be achieved [160]. Because ^166^Ho emits beta particles, the activated NPs were suggested as potential radiotherapeutic agents for the treatment of solid cancers. In a different study, ^198^Au-labeled gold NPs were labeled by neutron activation. In this case, thiol-functionalized gold NPs were activated by neutron irradiation at neutron flux of 10^14^ cm^−2^ s^−1^ at a research reactor. The amount of radioactivity was sufficient to approach subsequent* in vivo* studies using dissection and gamma counting. Importantly, the NPs were not altered by the neutron irradiation, despite the presence of organic functional groups on the surface of the gold core.

If the NPs to be investigated do not contain any atom susceptible to activation neither via neutron nor via ion, there is one alternative method, which can be assayed; the so-called recoil implantation. It relies on intimately mixing the NPs with a powder containing an atom, which can undergo a nuclear reaction under ion beam and hence act as a radiolabeling source. The high energy of the activated atom results in recoil implantation somewhere else, for example, in one of the NPs. This strategy has been used so far for the preparation of ^7^Be-labeled industrially manufactured SiO_2_ NPs [161].

## 5. Applications

IONP have emerged as a very interesting platform into which radionuclides and targeting moieties can be incorporated. This combination expands the field of application of IONP. Thanks to the hybrid nature of these particles, they can be used in PET/MRI and SPECT/MRI experiments [162, 163] or directly in PET/CT when MRI is no needed. In this sense, IONP can be used as a new kind of “chelate ligand” for radioisotopes but with size-dependent properties.

### 5.1. Biodistribution

IO-based nanoradiotracers have been reportedly tried* in vivo* in preclinical models to ensure biocompatibility and evaluate biodistribution. Devaraj et al. [43] synthesized cross-linked dextran-coated IONP radiolabeled with ^18^F via* click chemistry* and tried them in mice to determine biodistribution and blood clearance time of the NPs.

Stelter et al. [164] tried* in vivo*  ^68^Ga-DTPA-IONP in rats. PET and MR imaging determined hepatic and splenic accumulation of the NPs. The same NP accumulation trend was reported by Sharma et al. [48], who injected ^11^C-labeled IONP in healthy mice.

Glaus et al. [27] obtained IONP micelles radiolabeled with ^64^Cu via DOTA chelating agent and studied their biodistribution* in vivo *by PET and MRI in mice. Probe showed a circulation half-life of 143 min and hepatic and splenic accumulation 24 h after NP injection.

Sun et al. [44] radiolabeled comb-like oleylamine polyacrylic acid (COBP) IONP with ^18^F using NOTA.* In vivo *PET ad MR imaging in mice revealed hepatic and splenic NP uptake and no accumulation in bone.

**Figure 9:**
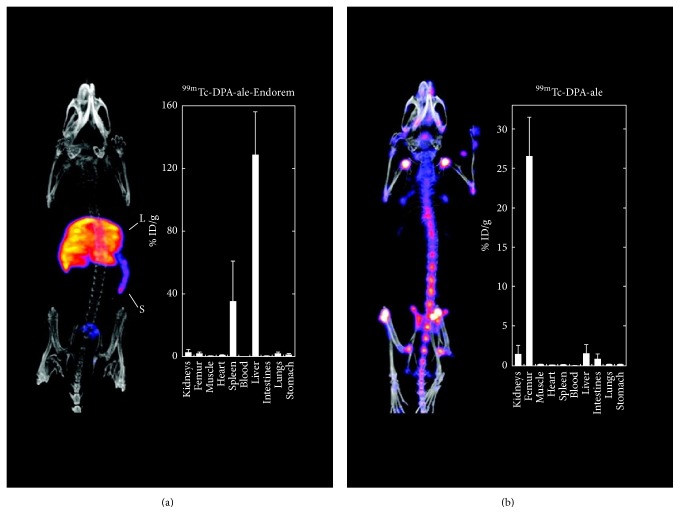
*In vivo* SPECT/CT maximum intensity projection (left) and biodistribution studies (right) of (a) ^99m^Tc-DPA-alendronate-Endorem and (b) ^99m^Tc-DPA-alendronate. Reproduced, with permission, from [22].

De Rosales et al. [22] radiolabeled Endorem (liver MRI contrast agent) with ^99m^Tc-DPA-alendronate (bisphosphonate SPECT agent), which bound directly the core of the IONP.* In vivo* SPECT and MR imaging revealed hepatic and splenic accumulation, meaning that the biodistribution of this complex is more similar to that of Endorem than to ^99m^Tc-DPA-alendronate, which accumulates in bone tissue (Figure 9).

Lee et al. [51] targeted asialoglycoprotein receptor in hepatocytes using lactobionic acid-functionalized ^99m^Tc-DTPA-IONP.* In vivo* SPECT in mice revealed hepatic NP accumulation.

### 5.2. Oncology

The availability of new and enhanced technologies allowing gene and protein expression study triggered immense progress in cancer biology and pharmacology, as well as clinical oncology [165]. Yet the absence of technology to study* in vivo* molecular events in depth has motivated the quest for novel approaches [166]. The combination of nanotechnology and radiochemistry, although quite novel, has already provided further enlightenment of cancer molecular mechanisms. It is not surprising that oncology is one of the main fields of research for these multimodal probes.

Aryal et al. [30] took advantage of the long circulating lifetime of their ^64^Cu-labeled PLGA-coated IONP clusters and the EPR effect in tumors to successfully locate breast cancer cells in mouse xenograft models using PET and* T*_2_-weighted MRI. Passive tumor accumulation strategy was used in another study carried out by Liu et al. [37] using PEG-coated ^64^Cu-radiolabeled MoS_2_ sheets containing IONP, which allowed tumor visualization and posterior tumor ablation by photothermal therapy in 4T1 tumor-bearing mice.

**Figure 10:**
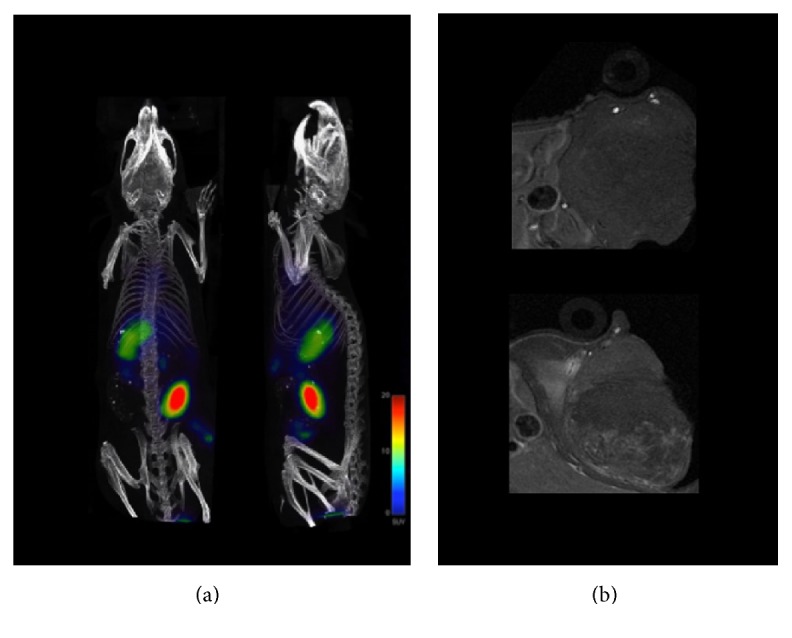
(a) PET/CT imaging of subcutaneous melanoma bearing mice 1 hour after injection of ^68^Ga-C-IONP-RGD. (b) Axial* T*_1_-weighted MRI of the tumor area in a mouse before the injection of ^68^Ga-C-IONP-RGD (top) and 24 hours after injection (bottom). Reproduced, with permission, from [23].

Lee et al. [26] labeled polyaspartic acid-coated IONP with ^64^Cu via DOTA chelator and conjugated them to RGD peptide to achieve targeted visualization of tumor integrin *α*_*v*_*β*_3_ expression in murine models bearing U87MG tumors, by both PET and MRI. RGD peptide was also chosen by Yang et al. [35] to direct SPIO nanocarriers, radiolabeled with ^64^Cu and containing doxorubicin, for tumor-targeted drug delivery and PET/MRI of U87MG-tumor-bearing mice. Deng et al. [53] used RDG-conjugated ^125^I-labeled IONP to target tumor cells for* in vivo* SPECT and MRI visualization in a breast cancer mouse model. Pellico et al. [23] obtained via microwave-assisted synthesis a chelator-free hybrid nanoradiotracer, ^68^Ga core-doped dextran-coated IONP, that was posteriorly conjugated to RGD peptide to target angiogenesis in a subcutaneous melanoma murine model.* In vivo *PET and* T*_1_-weighted MRI experiments confirmed specific tumor accumulation of the ^68^Ga-C-IONP-RGD probe (Figure 10).

Kim et al. [39] conjugated PEG-coated ^68^Ga-NOTA-IONP with oleanolic acid to specifically target HT-29 cells in a murine model and visualize tumor* in vivo *by PET and MRI.

To target prostate-specific membrane antigen (PSMA), Moon et al. [42] encapsulated IONP with amphiphiles containing PEG, DOTA, and PSMA-targeting ligand and radiolabeled them with ^68^Ga.* In vivo *PET and MR imaging experiments in mouse prostate cancer xenograft models revealed specific probe accumulation at tumor site.

Lymph nodes play a key role in cancer cell metastasis; for this reason numerous studies have focused on these structures to design novel hybrid probes for cancer staging. ^64^Cu-labeled IONP were synthesized by Torres Martin de Rosales et al. [32] to track lymph nodes* in vivo* in murine models. ^68^Ga-labeled IONP have been used in different studies to track lymph nodes using PET and MRI [38, 40, 145, 167]. Choi et al. [168] coated ^124^I-labeled IONP with serum albumin for the same purpose. Cross-linked PEG-coated IONP radiolabeled with ^124^I were selected by Park et al. [46] to track sentinel lymph nodes in murine 4T1 tumor xenograft model. Ferumoxytol was chosen by Thorek et al. [49] to synthesize a PET/MRI dual probe labeled with ^89^Zr via DFO chelator to track lymphatic drainage in murine diseased models. Cui et al. [169] reported synthesis and* in vivo *evaluation of Co_*x*_Fe_3−*x*_O_4_@NaYF_4_ core-shell NPs labeled with ^18^F to track lymph nodes in murine inflammation models. Chakravarty et al. [50] intrinsically labeled PEG-coated IONP with ^69^Ge to map lymph nodes in healthy mice. Madru et al. [52] used PEG-coated IONP labeled with ^99m^Tc and for imaging lymph nodes in rats using SPECT and MRI.

### 5.3. Cardiovascular Diseases

Cardiovascular diseases are the leading cause of death worldwide, accounting for more than 17 million deaths per year; a figure which is expected to keep rising in the coming years [170]. Most relevant advances in the treatment of these diseases have been focused on early diagnosis, for which molecular imaging is a key element.

Nahrendorf et al. [45] developed a multifunctional probe to detect macrophages in aortic aneurysms in using ^18^F-labeled cross-linked dextran-coated IONP.* In vivo *imaging experiments in murine models revealed specific probe accumulation in aneurysmatic aorta.

Ueno et al. [34] synthesized ^64^Cu-labeled IONP coated with cross-linked dextran to quantify infiltration of myeloid cells in mouse cardiac allografts.

Atherosclerotic plaque vulnerability to rupture is of paramount importance, as is it correlated to the risk of adverse coronary events. Dextran-coated IONP radiolabeled with ^64^Cu have been used in several studies to assess atherosclerotic plaque vulnerability targeting activated macrophages [29, 31, 33].

## 6. Conclusions

Nanomaterials in combination with radioisotopes are used more and more in molecular imaging. They show enhanced performance for hybrid imaging. Furthermore, it is possible to design them for tailored pharmacokinetics, for controlled biodistribution, and for the combination of diagnosis and therapy. In our opinion, new developments should focus on producing nanoradiomaterials showing features that are more intriguing than just the simple addition of their constituent parts. They should show synergistic behavior. Ideally, new features should appear from the combination of nanomaterials and radioisotopes. We have shown some of the most recent examples of this, but with the development of new materials and with their combination with different isotopes, the future of this combined approach seems promising both for preclinical imaging and for the patients.

## Figures and Tables

**Table 1:** Examples on the combined use of nanomaterials and radioisotopes.

Radioisotope	Nanomaterial	Radiolabeling method	Imaging techniques	Application	Reference
^64^Cu	Fe_3_O_4_-polyaspartic acid	Chelate approach (DOTA)	PET/MRI	Imaging of tumor integrin *α*_*v*_*β*_3_ expression	[26]
^64^Cu	*γ*-Fe_2_O_3_-polyethylene glycol	Chelate approach (DOTA)	PET/MRI	Biodistribution studies	[27]
^64^Cu	Fe_3_O_4_-dopamine-human serum albumin	Chelate approach (DOTA)	PET/MRI	U87MG tumor imaging	[28]
^64^Cu	Fe_3_O_4_-dextran	Chelate approach (DOTA)	PET/MRI	Cardiovascular plaque imaging	[29]
^64^Cu	Poly(lactic-co-glycolic) acid-Fe_3_O_4_-polyethylene glycol	Chelate approach (DOTA)	PET/MRI	Tumor imaging in breast cancer models	[30]
^64^Cu	Fe_3_O_4_-dextran	Chelate approach (DOTA)	PET/MRI	Activated macrophage detection in atherosclerotic plaques	[31]
^64^Cu	Fe_3_O_4_-dextran	Chelate approach (DTCBP)	PET/MRI	Lymph node imaging	[32]
^64^Cu	(Fe_2_O_3_)_*m*_(Fe_3_O_4_)_*n*_-dextran	Chelate approach (DTPA)	PET/MRI	Activated macrophage detection in atherosclerotic plaques	[33]
^64^Cu	(Fe_2_O_3_)_*m*_(Fe_3_O_4_)_*n*_-dextran	Chelate approach (DTPA)	PET/MRI	Myeloid cell detection in cardiac allografts	[34]
^64^Cu	Fe_3_O_4_-polyethylene glycol	Chelate approach (NOTA)	PET/MRI	Combined targeted anticancer drug delivery and tumor imaging	[35]
^64^Cu	Melanin-Fe-polyethylene glycol	Chelate approach (Melanin)	PET/MRI	Imaging of tumor integrin *α*_*v*_*β*_3_ expression	[36]
^64^Cu	Fe_3_O_4_-MoS_2_-polyethylene glycol	Chelate–free synthesis	PET/MRI	Combined photothermal therapy and imaging of tumors in breast cancer models	[37]
^68^Ga	Fe_3_O_4_-polyethylene glycol	Chelate-free synthesis	PET/MRI	Lymph node imaging	[38]
^68^Ga	Fe_3_O_4_-polyethylene glycol	Chelate approach (NOTA)	PET/MRI	Tumor imaging of HT-29 xenografts	[39]
^68^Ga	Fe_3_O_4_-polyethylene glycol	Chelate approach (NOTA)	PET/MRI	Lymph node imaging	[40]
^68^Ga	*γ*-Fe_2_O_3_-poly(lactic-co-glycolic) acid-b-polyethylene glycol	Chelate approach (NODA)	PET/MRI	Biodistribution studies	[41]
^68^Ga	Fe_3_O_4_-polyethylene glycol	Chelate approach (DOTA)	PET/MRI	PSMA-positive tumor imaging	[42]
^68^Ga	*γ*-Fe_2_O_3_-dextran	Core-doping approach	PET/MRI	Imaging of tumor integrin *α*_*v*_*β*_3_ expression	[23]
^18^F	(Fe_2_O_3_)_*m*_(Fe_3_O_4_)_*n*_-dextran	Click chemistry (copper-catalyzed azide-alkyne cycloaddition)	PET/MRI	Biodistribution studies	[43]
^18^F	Fe_3_O_4_-oleylamine branched polyacrylic acid	Chelate approach (NOTA)	PET/MRI	Biodistribution studies	[44]
^18^F	(Fe_2_O_3_)_*m*_(Fe_3_O_4_)_*n*_-dextran	Click chemistry (copper-catalyzed azide-alkyne cycloaddition)	PET/MRI	Macrophage detection in aortic aneurysms	[45]
^124^I	Fe_3_O_4_-polyethylene glycol	Surface labeling	PET/MRI	Lymph node imaging	[46]
^124^I	MnFe_2_O_4_-serum albumin	Surface labeling	PET/MRI	Lymph node imaging	[47]
^11^C	Fe_3_O_4_-COOH	Surface labeling	PET/MRI	Biodistribution studies	[48]
^89^Zr	Fe_3_O_4_-dextran	Chelate approach (DFO)	PET/MRI	Lymph node imaging	[49]
^69^Ge	Fe_3_O_4_-polyethylene glycol	Core-doping approach	SPECT/MRI	Lymph node imaging	[50]
^99m^Tc	Fe_3_O_4_-dextran	Chelate approach (DPA)	SPECT/MRI	Biodistribution studies	[22]
^99m^Tc	Fe_3_O_4_-dopamine-lactobionic acid	Chelate approach (DTPA)	SPECT/MRI	Liver imaging	[51]
^99m^Tc	Fe_3_O_4_-polyethylene glycol	Chelate approach (pertechnetate)	SPECT/MRI	Lymph node imaging	[52]
^125^I	Fe_3_O_4_-dextran	Chelate approach (CMD)	SPECT/MRI	Tumor imaging of breast cancer models	[53]
